# 5′-Nor-3-Deaza-1′,6′-Isoneplanocin, the Synthesis and Antiviral Study

**DOI:** 10.3390/molecules25173865

**Published:** 2020-08-25

**Authors:** Qi Chen, Stewart W. Schneller, Chong Liu, Kathryn L. Jones, Tyler Singer

**Affiliations:** 1 Department of Chemistry, Slippery Rock University, Slippery Rock, PA 16057, USA; klj1004@sru.edu (K.L.J.); tps1005@sru.edu (T.S.); 2 Molette Laboratory for Drug Discovery, Department of Chemistry and Biochemistry, Auburn, Auburn University, Auburn, AL 36849, USA; schnest@auburn.edu (S.W.S.); liuchon@auburn.edu (C.L.)

**Keywords:** antivirals, carbocyclic nucleosides, neplanocin, Ullmann reaction

## Abstract

The arbocyclic nucleosides aristeromycin and neplanocin have been studied as a source for new antiviral agents. A convenient synthesis of C-5′-truncated 3-deaza-1′,6′-isoneplanocin, which combines the features of antiviral candidates 5′-noraristeromycin and 3-deaza-1′,6′-isoneplanocin is reported from (−)-cyclopentenone to give the two C-4′ epimers of 5′-nor-3-deaza isoneplanocin. Antiviral assays showed activity against the JC virus (EC_50_ = 1.12 µM for (4′*R*)-**8**; EC_50_ = 59.14 µM for (4′*S*)-**7**) and inactivity of both compounds against several DNA and RNA viruses. Both compounds lacked cytotoxicity.

## 1. Introduction

Emerging and reemerging viral infectious diseases are continuously posing huge threats to global public health and have had a substantial socioeconomic impact. For example, a total of 28,616 confirmed and suspected cases with 11,310 deaths were reported during the 2014–2016 Ebola outbreak [1]. At the end of 2019, a novel coronavirus, named SARS-CoV-2, emerged and has infected 12,970,605 people in 188 countries/regions with 570,220 deaths (as of 13 July 2020 [2]) and continues to increase.

**Figure 1:**
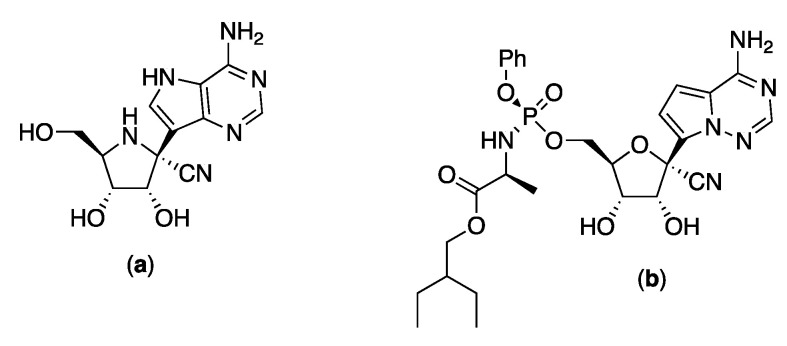
Examples of antivirals as nucleosides and nucleotides analogues: (**a**) BCX 4430 (Galidesivir); (**b**) GS-5734 (Remdesivir).

In the search for antiviral countermeasures, repurposed or newly designed nucleosides and nucleotide analogues are serving as a resource for the frontline defense, especially in those urgent situations [3,4]. For instance, BCX 4430 (Galidesivir, **a**) and GS-5734 (Remdesivir, **b**) (Figure 1) were developed during the 2014–2016 Ebola outbreak [5]. Because of its activity towards SARS-CoV-2, Remdesivir is being repurposed for treatment in this current pandemic.

**Figure 2:**
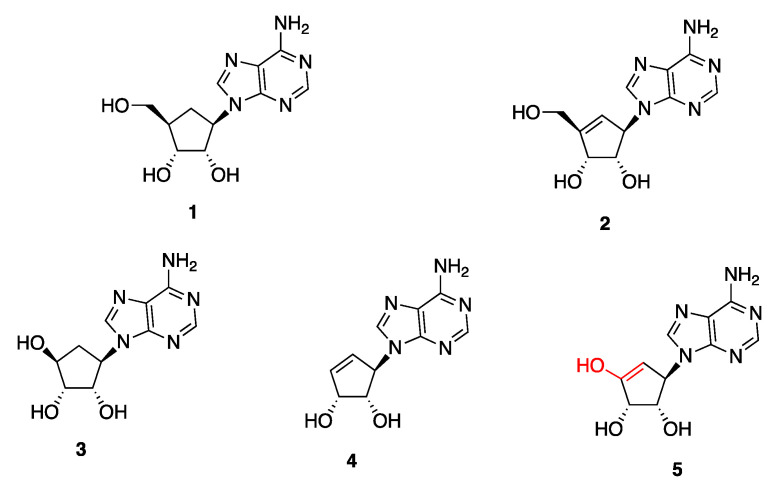
Structures of carbocyclic nucleosides: **1**. Aristeromycin; **2**. Neplanocin; **3**. 5′-noraristeromycin; **4**. 4′-deoxymethylene neplanocin; **5**. 5′-norneplanocin.

Galidesivir (**a**) and Remdesivir (**b**) are C-nucleosides with the glycosidic linkage replaced by a more stable C-C bond and, hence, are metabolically stable to hydrolytic and phosphorlytic breakdown, a relevant feature for nucleoside-based therapeutic candidates [6]. A similar property is seen with carbocyclic nucleosides, such as the naturally occurring aristeromycin (**1**) and neplanocin A (**2**), (Figure 2) which possess antibacterial, -parasitic, -viral and -cancer properties [3,7], due, principally, to the non-selective inhibition of *S*-adenosylhomocysteine hydrolase (SAHase). The therapeutic of **1** and **2** is limited by their cytotoxicity as a result of biomolecular inference by their 5′-phosphate metabolites.

To address this undesirable feature, the C-4′ truncated variations (**3** and **4**) were prepared and found to be effective against a number of viruses and to be non-cytotoxic [8]. A similar modification on neplanocin A (that is, **5**) is, however, unlikely due to its enolic structure (red structure in Figure 2).

**Figure 3:**
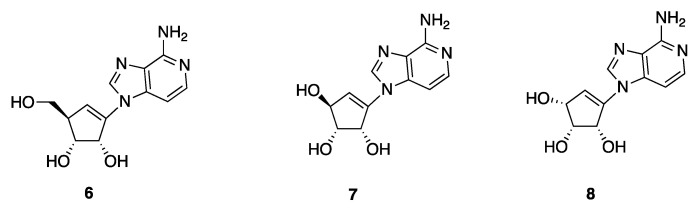
Isoneplanocin analogues and designed target compounds: **6**. 3-deaza-isoneplanocin; **7**. (4′*S*)-3-deaza-5′-norisoneplanocin; **8**. (4′*R*)- 3-deaza-5′-norisoneplanocin.

Another carbocyclic nucleoside structural modification developed in our labs has been the 1′,6′-isoneplanocin series (herein designated as isoneplanocin and represented by the 3-deaza analogue, **6**) that displays a broad-based, non-cyctotoxic antiviral profile [9]. We have recently desired to combine the features of **3** with **6** and, thus, set **7** and **8** as targets. (Figure 3) These results are reported here.

## 2. Results

**Scheme 1:**
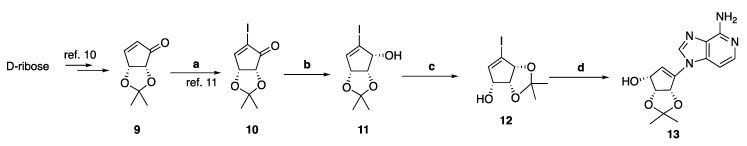
Reagents and conditions: (**a**) I_2_, pyridine, CCl_4_, rt., 3 h, 90%; (**b**) NaBH_4_, CeCl_3_^.^7H_2_O, MeOH, rt., 2 h, 93%; (**c**) *p*-TsOH, acetone, rt., overnight, 48% (50% recovered **11**); (**d**) 3-deazaadenine, K_2_CO_3_, dipivaloylmethane, CuI, 120 °C, overnight, <5%.

Ullmann coupling of a vinyl iodide with an adenine moiety is well established in our lab as a powerful synthetic tool for the preparation of 1′,6′-isoneplanocin analogues [9]. For the purposes of this investigation, vinyl halide **11** was foreseen as the requisite building block. Its synthesis (Scheme 1) began with the iodination of protected (−)-cyclopentenone **9**, available from ribose [10,11], to **10**. Luche reduction of **10** to allylic alcohol **11**, which, upon acid catalyzed isopropylidene rearrangement was expected [12] to provide **12** but resulted in an inseparable mixture with unreacted **11**. As a consequence, this mixture was subjected to the Ullmann conditions with 3-deazaadenine [13], and a low yield of **13** (that is, its protected form, **8**) occurred.

**Scheme 2:**
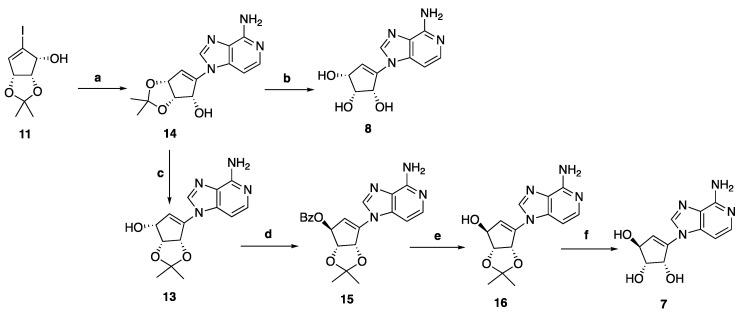
Reagents and conditions: (**a**) 3-deazaadenine, K_2_CO_3_, dipivaloylmethane, CuI, 120 °C, overnight, 51%; (**b**) 2 M HCl/MeOH, rt., 1 h, 85%; (**c**) HCl, MeOH, **13**, 42%; (56% recovered **14**); rt., overnight. (**d**) Ph_3_P, DIAD, benzoic acid, THF, rt., 12 h, 65%; (**e**) LiOH, THF-H_2_O (1:1), rt., 6 h, 95%; (**f**) HCl, MeOH, rt., overnight, 90%.

Our attention turned to employing the Ullmann coupling of **11** and 3-deazaadenine. This succeeded in giving **14** (Scheme 2) in a moderate yield in contrast to **12**, suggesting a hydroxyl substituent adjacent to the vinyl coupling site was necessary for the Ullmann to succeed. Acid deprotection of **14** availed the desired (4′*R*)-8. In addition to NMR data, the structure of **8** was confirmed by X-ray crystallography (CCDC 2018731), which served to confirm the regiochemistry of the cyclopentenyl and the 3-deaza base of **8** (Supplementary Meterials).

To achieve epimer **7**, acid catalyzed isopropylidene rearrangement of **14** to **13** was followed by a Mitsunobu C-4′ inversion to **15**. Basic removal of the benzoate of **15** to **16** and subsequent acid deprotection yielded (4′*S*)-7.

## 3. Discussion

Compounds **7** and **8** were subjected to antiviral assays [14]. Compound **8** displayed potent activity (EC_50_ = 1.12 μM) against the JC virus, a polyomavirus. Compound **7** had much lower activity (EC_50_ = 59.14 μM) against the JC virus. Both epimers showed no cytotoxicity (CC_50_ > 150 μM) towards the host COS7 cell-line. There was no activity for either compound against human cytomegalovirus, adenovirus, vaccinia virus, Epstein–Barr virus and human norovirus. No cytotoxicity was found as a result of these assays.

Further studies will consider variations of **8** for improving its JC antiviral potential, correlating its enzymatic effects (for example, towards SAHase) with the parent **6**, and its usefulness for developing novel C-4′ hydroxyl-based analogues within the 3-deazaisoneplanocin series.

## 4. Materials and Methods

### General Procedure of Ullmann Reaction

Vinyl iodide (1 mmol) was dissolved in DMSO (10 mL) under N_2_. 3-Deazaadenine (1.25 mmol), K_2_CO_3_ (117 mg), dipivaloylmethane (DPM) (27 µL) and CuI (13 mg) were added in sequence. The reaction was heated to 120 °C in an oil bath overnight. The solvent was evaporated under vacuum and the residue was purified by column chromatography (EtOAc:hexanes = 1:1).

(1S,2R,3S)-4-(4-amino-1H-imidazo[4,5-c]pyridin-1-yl)cyclopent-4-ene-1,2,3-triol ((4′S)-7): ^1^H NMR (500.3 MHz, D_2_O) δ 8.42 (s, 1H), 7.61 (d, *J* = 7.0 Hz, 1H), 7.23 (d, *J* = 7.0 Hz, 1H), 6.26 (d, *J* = 2.0 Hz, 1H), 5.03 (m, 1H), 4.85 (m, 1H), 4.11 (t, *J* = 5.0 Hz, 1H); ^13^C NMR (125.8 MHz, D_2_O) δ 151.3, 141.5, 140.8, 139.2, 138.3, 126.3, 121.2, 100.1, 71.8, 71.5, 70.5. Analogue was calculated for C_11_H_12_N_4_O_3_: C, 53.22; H, 4.87; N, 22.57. Found: C, 53.01; H, 4.94; N, 22.29.

(1R,2R,3S)-4-(4-amino-1*H*-imidazo[4,5-c]pyridin-1-yl)cyclopent-4-ene-1,2,3-triol ((4′R)-8): ^1^H NMR (500.3 MHz, DMSO-*d*_6_) δ 8.34 (s, 1H), 7.76 (d, *J* = 6.0 Hz, 1H), 7.31 (d, *J* = 6.0 Hz, 1H), 6.27(s, 2H), 6.13 (d, *J* = 2.0 Hz, 1H), 5.13 (d, *J* = 8.0 Hz, 1H), 4.84 (m, 2H), 4.54 (d, *J* = 7.5 Hz, 1H), 4.49 (m, 1H), 4.12 (m, 1H). ^13^C NMR (125.8 MHz, DMSO-*d*_6_) δ 152.6, 141.7, 139.8, 139.5, 137.0, 126.4, 118.6, 98.1, 71.7, 71.3, 70.0. HRMS (ESI) was calculated for C_11_H_13_N_4_O_3_: 249.0988. Found (M + H)^+^ 249.0987.

## Supplementary Materials

The following are available online. Figure S1: 1HNMR spectrum of 7, Figure S2: 13CNMR spectrum of 7, Figure S3: 1HNMR spectrum of 8, Figure S4: 13CNMR spectrum of 8, Figure S5: X-ray crystallography of 8, crystallographic data (excluding structure factors) is available in the Cambridge Crystallographic Data Centre, CCDC 2018731.
