# Supplementary material for: 5′-Nor-3-Deaza-1′,6′-Isoneplanocin, the Synthesis and Antiviral Study
Source: Molecules. 2020 Aug 25;25(17):3865. doi: 10.3390/molecules25173865 (PMC7503852; doi:10.3390/molecules25173865)
Supplement: Supplementary file 1 [file molecules-25-03865-s001.pdf]

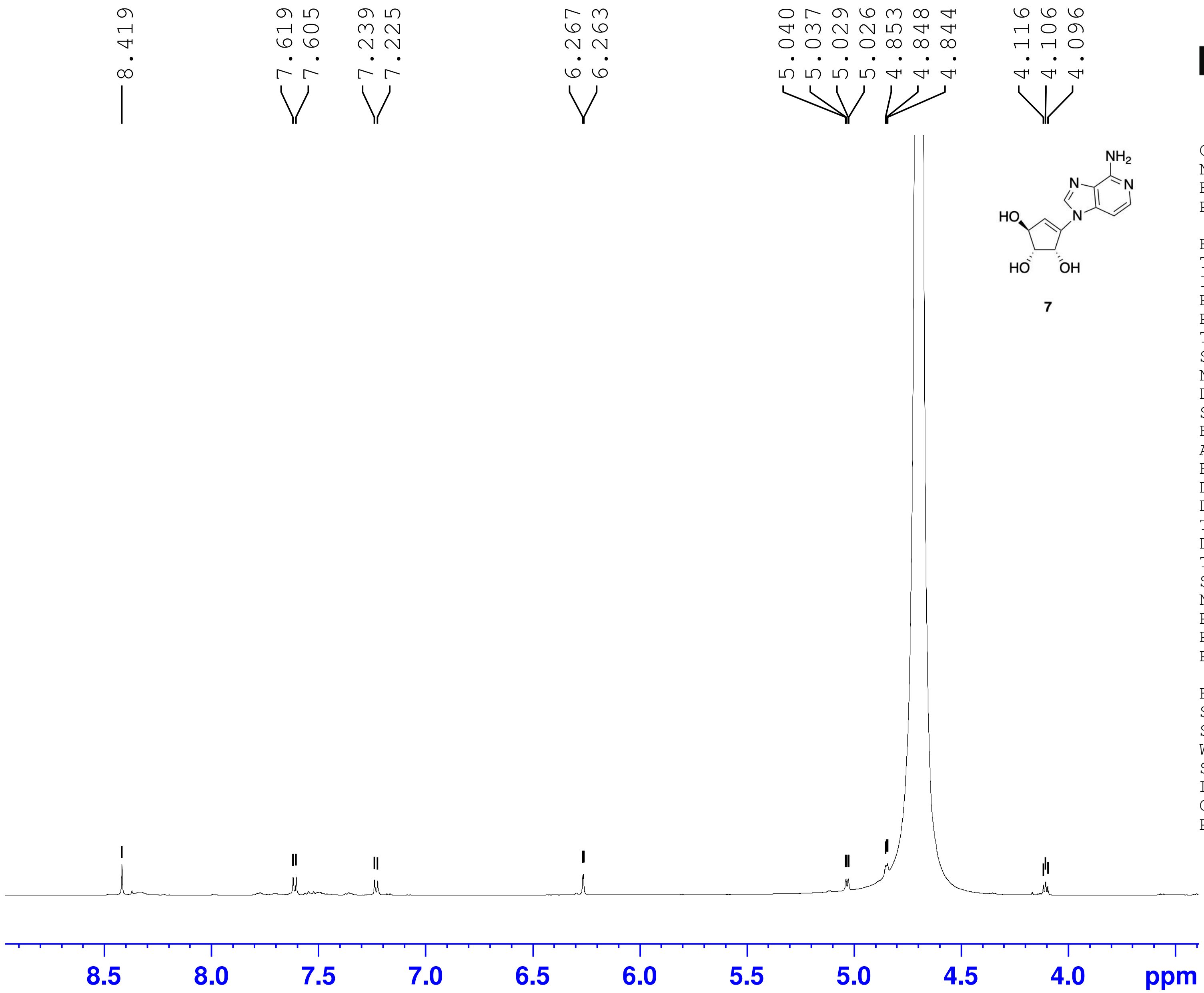

7

Current Data Parameters
NAME QC19118
EXPNO 1
PROCNO 1

F2 - Acquisition Parameters
Time 16.45 h
INSTRUM Avance Neo 500
PROBHD Z168772_0004 (
PULPROG zg30
TD 65536
SOLVENT D2O
NS 16
DS 2
SWH 10000.000 Hz
FIDRES 0.305176 Hz
AQ 3.2767999 sec
RG 12.7141
DW 50.000 usec
DE 10.45 usec
TE 298.1 K
D1 1.00000000 sec
TD0 1
SFO1 500.3230895 MHz
NUC1 1H
P0 4.00 usec
P1 12.00 usec
PLW1 12.83399963 W

F2 - Processing parameters
SI 65536
SF 500.3200000 MHz
WDW EM
SSB 0
LB 0.30 Hz
GB 0
PC 1.00

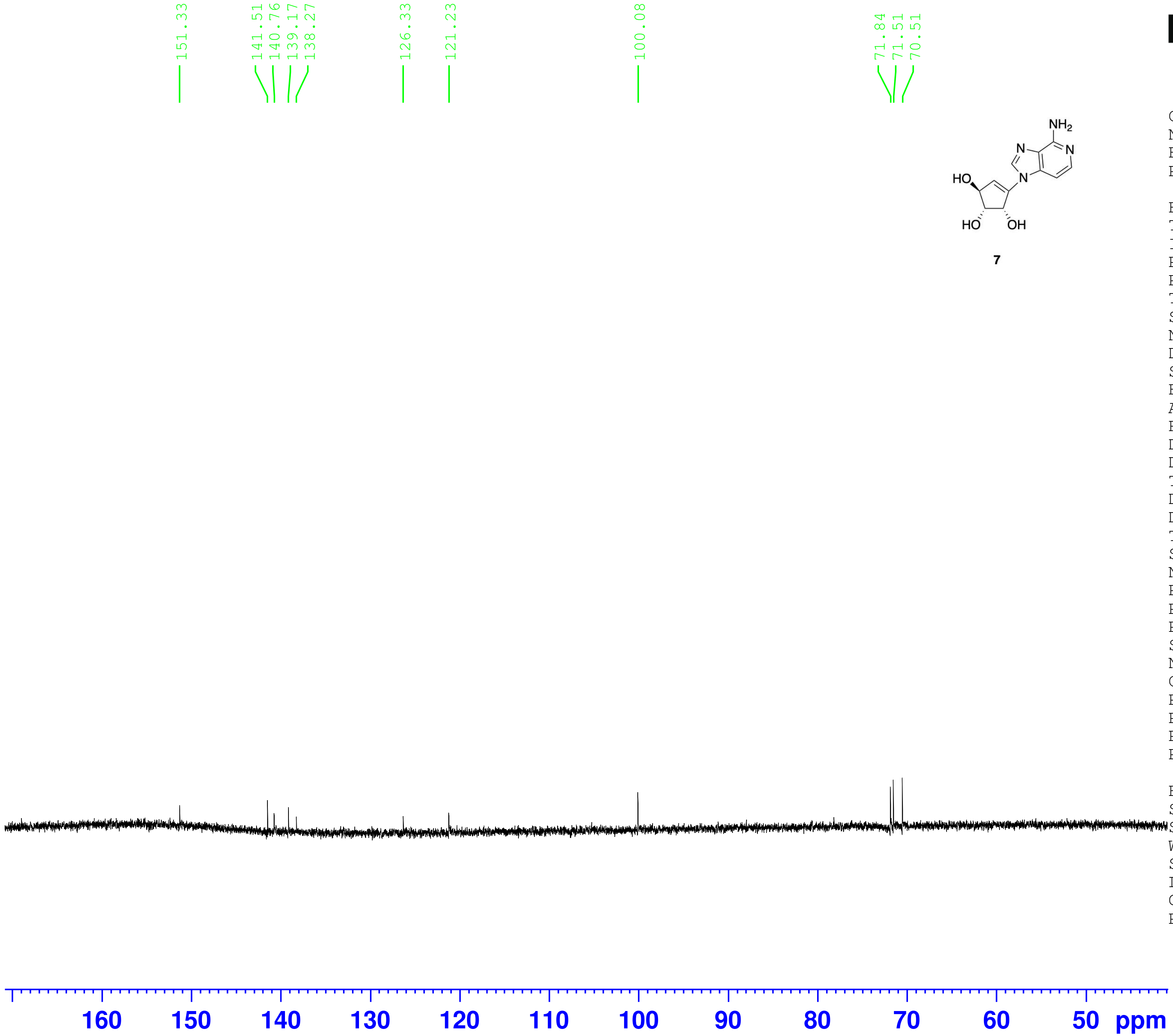

7

Current Data Parameters
NAME QC1911-8
EXPNO 3
PROCNO 1

F2 - Acquisition Parameters

Time	10.09	h
INSTRUM	Avance Neo 500	
PROBHD	Z168772_0004 (	
PULPROG	zgpg30	
TD	65536	
SOLVENT	D2O	
NS	1024	
DS	4	
SWH	30120.482	Hz
FIDRES	0.919204	Hz
AQ	1.0878977	sec
RG	101	
DW	16.600	usec
DE	18.00	usec
TE	298.2	K
D1	2.00000000	sec
D11	0.03000000	sec
TD0	1	
SFO1	125.8181446	MHz
NUC1	13C	
P0	3.33	usec
P1	10.00	usec
PLW1	58.06100082	W
SFO2	500.3220013	MHz
NUC2	1H	
CPDPRG[2	waltz65	
PCPD2	80.00	usec
PLW2	12.83399963	W
PLW12	0.28833300	W
PLW13	0.14451250	W

F2 - Processing parameters

SI	32768	
SF	125.8055641	MHz
WDW	EM	
SSB	0	
LB	1.00	Hz
GB	0	
PC	1.40	

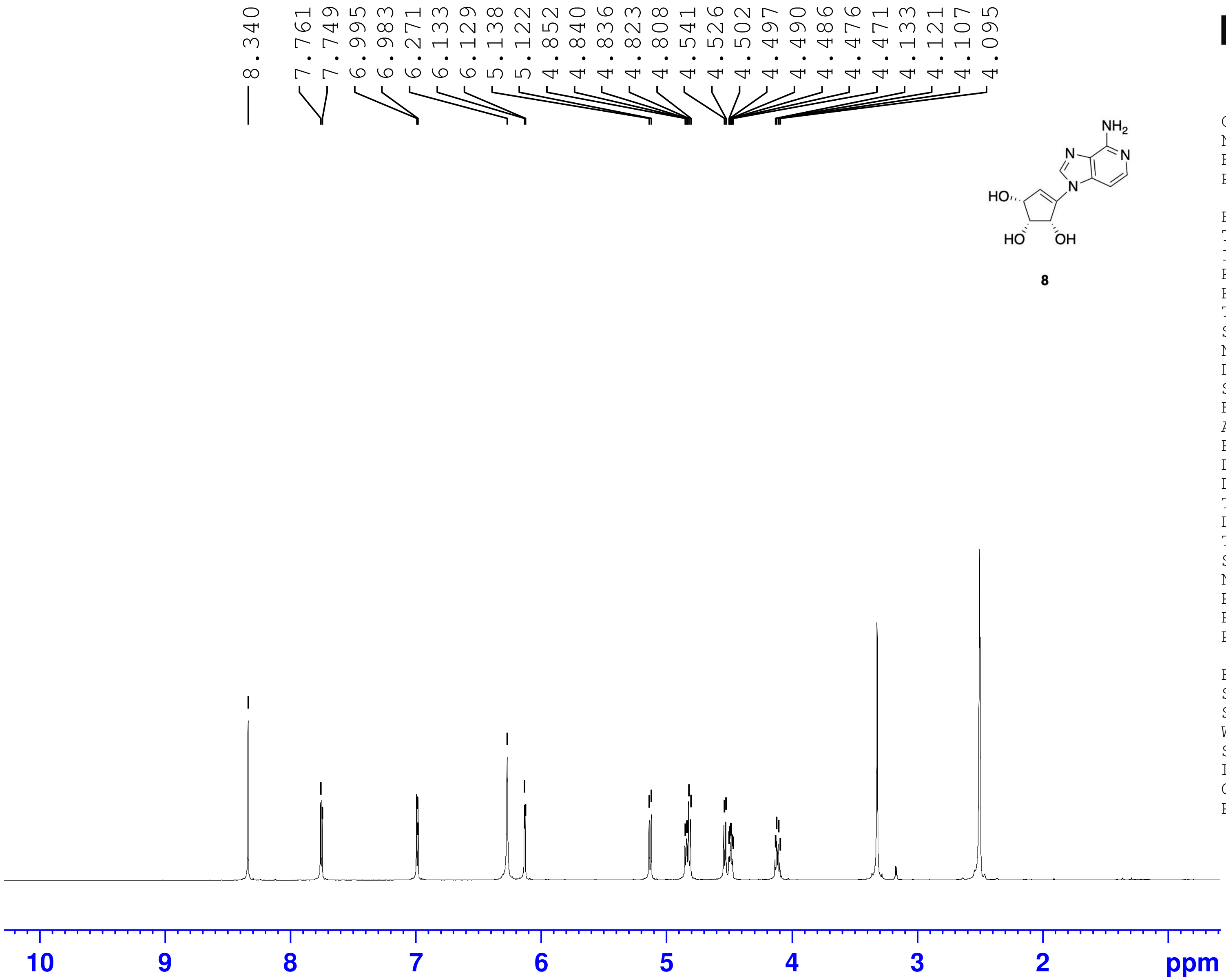

8

```
Current Data Parameters
NAME              QC198-11
EXPNO                    6
PROCNO                   1

F2 - Acquisition Parameters
Time                 10.18 h
INSTRUM     Avance Neo 500
PROBHD      Z168772_0004 (
PULPROG               zg30
TD                   65536
SOLVENT               DMSO
NS                      16
DS                       2
SWH              10000.000 Hz
FIDRES            0.305176 Hz
AQ               3.2767999 sec
RG                 61.1765
DW                  50.000 usec
DE                   10.45 usec
TE                   298.1 K
D1              1.00000000 sec
TD0                      1
SFO1           500.3230895 MHz
NUC1                    1H
P0                    4.00 usec
P1                   12.00 usec
PLW1           12.83399963 W

F2 - Processing parameters
SI                   65536
SF             500.3200038 MHz
WDW                     EM
SSB                      0
LB                    0.30 Hz
GB                       0
PC                    1.00
```

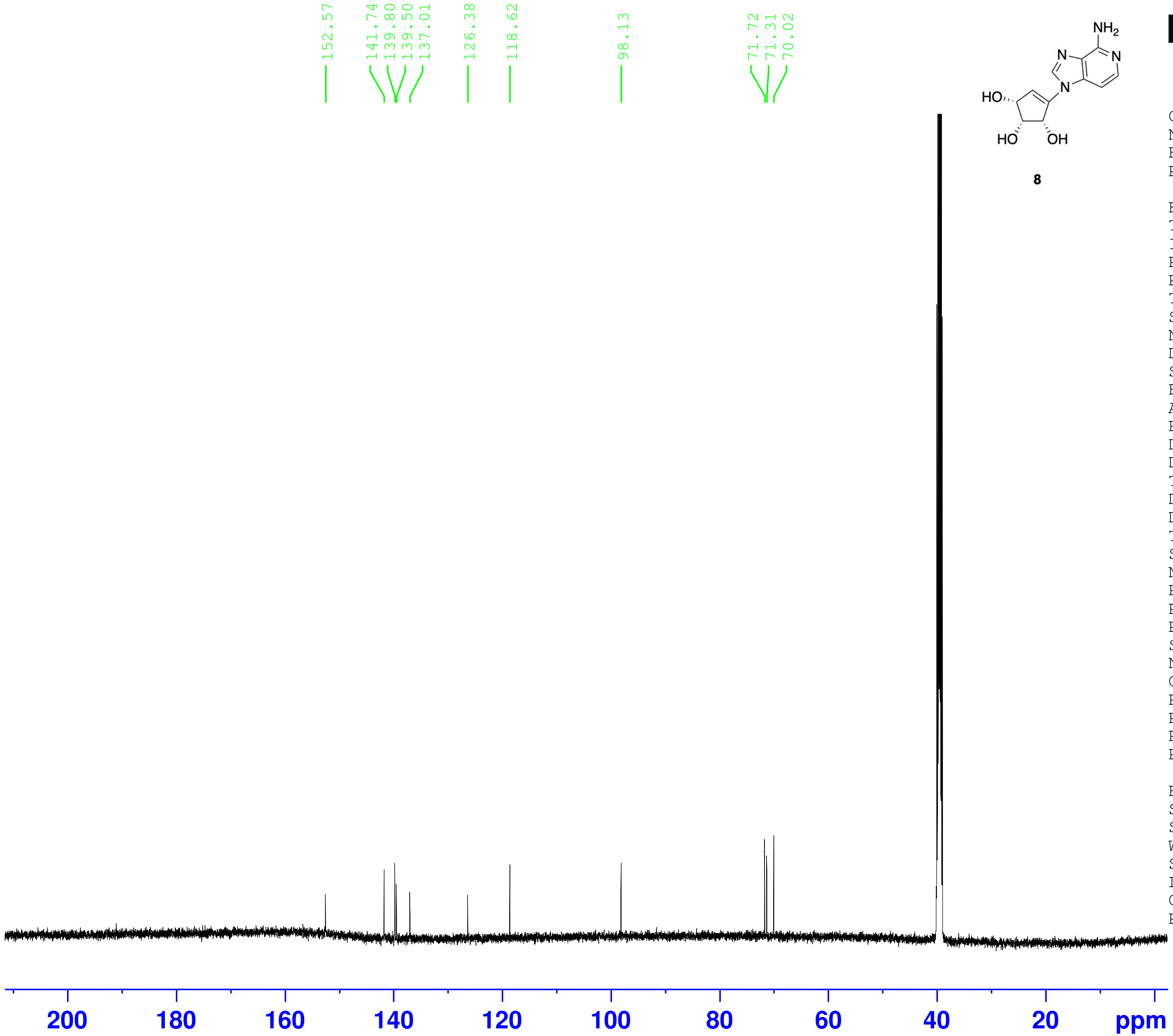

8

Current Data Parameters
NAME QC198-11
EXPNO 7
PROCNO 1

F2 - Acquisition Parameters
Time 10.45 h
INSTRUM Avance Neo 500
PROBHD Z168772_0004 (
PULPROG zgpg30
TD 65536
SOLVENT DMSO
NS 442
DS 4
SWH 30120.482 Hz
FIDRES 0.919204 Hz
AQ 1.0878977 sec
RG 101
DW 16.600 usec
DE 18.00 usec
TE 298.2 K
D1 2.00000000 sec
D11 0.03000000 sec
TD0 1
SFO1 125.8181446 MHz
NUC1 13C
P0 3.33 usec
P1 10.00 usec
PLW1 58.06100082 W
SFO2 500.3220013 MHz
NUC2 1H
CPDPRG[2 waltz65
PCPD2 80.00 usec
PLW2 12.83399963 W
PLW12 0.28833300 W
PLW13 0.14451250 W

F2 - Processing parameters
SI 32768
SF 125.8056293 MHz
WDW EM
SSB 0
LB 1.00 Hz
GB 0
PC 1.40

X-ray crystallography data for compound 8

Crystallographic data (excluding structure factors) for the structure in this paper have been deposited with the Cambridge Crystallographic Data Centre as supplementary publication number CCDC 2018731. Copies of the data can be obtained, free of charge, on application to CCDC, 12 Union Road, Cambridge CB2 1EZ, UK, (fax: +44 1223 336033 or e mail: deposit@ccdc.cam.ac.uk)

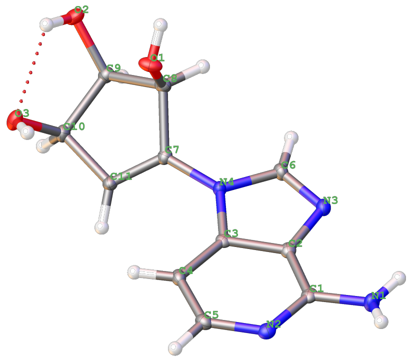

(3a*R*,4*R*,6a*S*)-6-(4-amino-1*H*-imidazo[4,5-c]pyridin-1-yl)-2,2-dimethyl-3a,6a-dihydro-4H-cyclopenta[d][1,3]dioxol-4-ol (13)

^{1}HNMR (400 MHz, $CDCl_3$) *δ* 8.25 (s, 1H); 7.95 (d, *J* = 6.0 Hz, 1H); 6.94 (d, *J* = 6.0 Hz, 1H); 6.04 (d, *J* = 1.2 Hz, 1H); 5.40 (d, *J* = 5.6, 1H); 5.26 (br, 2H); 4.96 (t, *J* = 5.6 Hz, 1H); 4.85 (dd, *J* = 5.6, 1.6 Hz, 1H); 1.50 (s, 3H); 1.47 (s, 3H).

(3a*S*,4*S*,6a*R*)-5-(4-amino-1*H*-imidazo[4,5-c]pyridin-1-yl)-2,2-dimethyl-3a,6a-dihydro-4*H* cyclopenta[d][1,3]dioxol-4-ol (14)

^{1}HNMR (400 MHz, $CDCl_3$) *δ* 8.35 (s, 1H); 7.95 (d, *J* = 6.0 Hz, 1H); 6.94 (d, *J* = 6.0 Hz, 1H); 6.07 (d, *J* = 2.0 Hz, 1H); 5.27 (dd, *J* = 5.6, 2.0 Hz, 1H); 5.20 (br, 2H); 5.01 (d, *J* = 5.6 Hz, 1H); 4.94 (t, *J* = 6.0 Hz, 1H); 1.52 (s, 3H); 1.49 (s, 3H). ^{13}CNMR (100 MHz, $CDCl_3$) *δ* 152.1, 142.7, 140.6, 140.0, 138.1, 115.4, 113.2, 99.5, 81.0, 77.4, 75.9, 72.6, 28.0, 26.7.

(3aR,4S,6aS)-6-(4-amino-1H-imidazo[4,5-c]pyridin-1-yl)-2,2-dimethyl-3a,6a-dihydro-4H-cyclopenta[d][1,3]dioxol-4-yl benzoate (15)

To a solution of ph_3P (262 mg, 1.0 mmol) and DIAD (202 g, 1.0 mmol) in anhydrous THF (5 mL) were added benzoic acid (110 mg, 1.0 mmol) and a solution of compound **13** (200 mg, 0.69 mmol) in THF (5 mL) at 0°C under nitrogen atmosphere. Then the reaction was brought to room temperature and stirred at same temperature for 12 hours. The solvent was evaporated under reduced pressure and resulting residue was purified by column chromatography to give **15** contaminated with triphenylphosphine oxide (176 mg, 65%).

^{1}HNMR (400 MHz, $CDCl_3$) *δ* 8.30 (s, 1H); 8.06 (m, 2H); 7.94 (d, *J* = 6.0 Hz, 1H); 7.53 (m, 3H); 6.96 (d, *J* = 6.0 Hz, 1H); 6.18 (d, *J* = 2.8 Hz, 1H); 5.99 (m, 1H); 5.71 (dd, *J* = 6.0, 1.2 Hz, 1H); 5.53 (br, 2H); 4.96 (d, *J* = 6.0 Hz, 1H); 1.48 (s, 3H); 1.47 (s, 3H).

(3a*R*,4*S*,6a*S*)-6-(4-amino-1*H*-imidazo[4,5-c]pyridin-1-yl)-2,2-dimethyl-3a,6a-dihydro-4*H*-cyclopenta[d][1,3]dioxol-4-ol (16)

To the crude **15** (176 mg, 0.45 mmol) was treated with LiOH (68 mg mg, 1.35 mmol) in THF-H_2O (1:1) solution (8 mL) for 12 hours at room temperature. The solvent was evaporated under reduced pressure and resulting residue was purified by column chromatography to give **16** contaminated with tripenylphosphine oxide (127 mg, 95%).

^{1}HNMR (400 MHz, $CDCl_3$) *δ* 8.28 (s, 1H); 7.78 (m, 1H); 6.93 (m, 1H); 6.05 (m, 1H); 5.64 (d, *J* = 5.2 Hz, 1H); 4.97 (s, 1H); 4.72 (d, *J* = 5.6 Hz, 1H); 1.42 (s, 6H).
